# Elements of Pathological Anatomy; Illustrated by Numerous Engravings

**Published:** 1840-10

**Authors:** 


### REVIEWS.

*(Continued from the September number.)*

Art. III.—*Elements of Pathological Anatomy;* illustrated by numerous engravings. By Samuel D. Gross, M. D. Boston: 1839.

The notices of some of the first chapters this book contained in our last number were a part of the article published in the preceding, the limits of which did not permit an insertion of the whole. The division was made in the absence of the Reviewer, and the article appeared, therefore, without exordium or peroration. With this explanation we proceed to the analysis of other portions of this comprehensive work.

The 15th chapter treats of hydatids. We agree with our author when he affirms, that most American physicians are unacquainted with this subject. It is but justice, however, to add, that, curious and interesting in a physiological and zoological point of view, as these productions may be, their study by the practical physician, is not of very great importance. This results, first from the rarity of hydatids in the *human* body; and second, from the impossibility in most cases of relieving the patient, when they are known to exist. Nevertheless, every physician ought to have a general knowledge of this class of beings; and such a knowledge can be successfully acquired in a single hour by the study of the dozen pages which our author has devoted to them.

"Hydatids occur in serous cavities, the alimentary canal and the passages which open into it, the cellular tissue, between the muscles, and in the proper substance of the different organs. Nevertheless, there are, as will be seen hereafter, some parts that are more frequently affected than others. They have been found in nearly all classes of animals,—in birds, reptiles, and fishes, as well as in a great many of the mammalia. Whether they exist in insects, is a point which has not been ascertained. No period of life is exempt from them. Portal, indeed, mentions an instance of their having been detected in the fœtus. They are most common, however, in adults and old people.

"So far as can be ascertained, these parasitic beings possess no genital organs, no apparatus for respiration, no trace of a circulation, and apparently no nerves. They can live and propagate their species only in the interior of other animals, and their existence is usually very brief, most of them perishing within the first year or two after they are developed, often much earlier. A few of them only are capable of performing distinct movements, under the influence of external stimulants. The cysticercus, for example, when put in luke-warm water, not only whirls itself about, but alternately protudes and retracts its suckers. The acephalocyst, on the contrary, remains perfectly quiescent, and may therefore be said to be void of irritability and contractility."

Hydatids are naturally divisible into two great classes—the cephalocysts, or those in which a head is associated with the cyst, and acephalocysts, or those in which there is no appearance of such an organ or any other. Of the former our author describes and figures four genera: 1st. The *cysticercus*, or bladder-tailed hydatid, of which he enumerates, with Cloquet, five species; most of which have been found but a single time in the human body, all of them in the plexus choroides, and some of them there only. In our domestic animals, as the hog, sheep, ox, and goat, they are more common. 2d. The *polycephalus*, or many-headed hydatid, not yet found in man. 3d. The *diceras*, or two-headed hydatid, observed, once, to have been discharged from the human bowels. 4th. The *echinococcus*, or rough hydatid, found a single time in the brain, and discharged once from the bladder.

From this enumeration it will be perceived, that the cephalocystic hydatids do not present themselves often enough in the human subject to be of much interest. Their greater frequency, in our domestic animals, constitutes them legitimate objects of epizootic medicine.

The other great class, *acephalocysts*, are much more common in the human body, and therefore more deserving of notice.

"Occurring both in the human subject and in many of the inferior animals, the individuals of this class of parasites infest some organs much more frequently than others. They seem to have a remarkable predilection for the liver, owing, probably, to some peculiarity of structure favoring their development. The brain, ovary, uterus, mammary gland, spleen, and kidney, are also sometimes their seat; in fact, they have been found in every part of the body, except the alimentary canal, the urinary bladder, and the respiratory passages.

"Varying in size between a mustard-seed and a large orange, they are generally of a spherical figure, and composed of a white, semi-opaque, pulpy vesicle, filled with a clear, limpid fluid. This vesicle, which forms the hydatid, properly so called, is from the sixth of a line to the eighth of an inch in thickness, and is often separable into two or more layers, and is so exceedingly delicate as to yield under the slightest pressure of the finger. So weak is it, indeed, that it is frequently incapable of withstanding the pressure of its own contents, as I have had repeated opportunities of witnessing, after the partial removal of the enclosing cyst. On being ruptured, it shrinks up into a soft, irregular, pulpy mass, of an opaline color, which readily swims in water, and bears the greatest resemblance to coagulated white of egg.

"It sometimes happens, though not very often, that a large acephalocyst contains several that are smaller, one within the other, all of the same shape and structure. As many as three, four, and even five, have been found thus enclosed, like so many pill-boxes. This arrangement, which occurs much oftener in the human subject than in the inferior animals, is explained by the endogenous mode of generation previously adverted to, by which one acephalocyst, after having arrived at maturity, produces another, each successive one being smaller than its parent."

Our author like most of his predecessors indulges but little in speculations on the origin of hydatids; yet true to the theory, that most morbid products are the result of inflammatory action he strongly inclines to that view of their origin. In speaking of the causes which develope acephalocysts, he remarks:

"In Cincinnati, where there are annually slaughtered upwards of one hundred thousand hogs, probably not a tenth part are free from this disease. Whole droves, consisting of three or four hundred head, are sometimes thus affected. These animals, most of which are young, are raised in the prairie districts of Ohio, Indiana and Kentucky, and are literally stuffed, for six or eight weeks before being sent to market, with fresh corn. The consequence is, that the portal circle is kept in a state of constant congestion, which finally leads to inflammatory irritation and the development of acephalocysts in the liver and other viscera. The irritation thus set up is of a specific nature, and is followed by the deposition of a fibro-albuminous substance, or, what is the same thing, a sort of plastic lymph, the particles of which arrange themselves in such a manner as to create an inferior being, an entozoic parasite."

Undoubtedly, the great hydatid development in these cases is connected with an increase in the diet of the animal, originating a very active and nutritive process, particularly the adipous system; but we do not admit, that such a condition is necessarily attended with inflammation. In fact, we generally find, that individuals who generate great quantities of fat are less than others inclined to the *phlegmasiæ*, for the secretion of so much fat keeps down plethora. But if excess of nourishment generates hydatids by exciting inflammatory action, how does it happen, as we state in the words of our author, that in many, ruminants, hydatids may be produced almost at pleasure, by confining them in moist situations, and restricting them to very juicy unripe vegetables? Is this a regimen, which is likely to raise inflammation? Under both modifications of diet we see causes which are calculated to affect the nutritive and secretory functions, and nothing more. If these causes excite inflammation, that state cannot explain the development of hydatids; inasmuch as there is no necessary connection, between the state of an inflammed part and the formation in it of an entozoa. If inflammation could give birth to hydatids, how does it happen, that we do not find them as frequently, as we find the acknowledged products of that pathological condition? Should an organ be at the same time affected with hydatids and inflammation, it would not follow that the latter was the cause of the former. It would be less gratuitous to affirm the reverse, and assign the hydatids as the cause of the inflammation. The origin of hydatids and all other entozoa, not known to exist out of the living body, presents us with a problem in transcendental physiology; but the remote causes which favor their development, are legitimate objects of study, and should be ascertained that they may be avoided.

Chapter xvi. treats of serous cysts. These productions bear a marked resemblance to the second great division of hydatids—the acephalocystic. They differ, however, from the latter, in being always attached to the adjoining normal tissues, which supply them with blood vessels. They are sometimes solitary and simple, that is consisting of a single cavity; at other times, this cavity is subdivided, by processes of the membrane which constitutes its parietes, and it then becomes multilocular; in which case the different cells may contain fluids very unlike each other, as serum, pus, blood, fat, a honey like substance, &c. Finally, a great number of simple cysts may be aggregated in one cavity, attached directly to its parietes, or indirectly by being joined to each other.

"It will be recollected that these different classes of cysts are the result, in most instances, of an entirely new formation, dependent upon a perverted state of the nutritive function. In other cases, they appear to be formed out of preexisting textures, sometimes of a serous, at other times of a mucous nature. To the former category belong the cysts, which are so often found in the ovaries, in consequence of the enlargement of the vesicles of De Graaf; to the latter, those which are developed in the kidneys and in the female breasts, from obstruction of the excretory duct. In these situations it is not uncommon for the adventitious growth to receive an accidental covering from the organ in which it is located. In the ovaries, for example, we accordingly find that the cyst is usually provided with very thick, dense parietes, separable into three distinct layers, the internal of which consists of the capsule of the vesicle of De Graaf, the second of the albugineous, and the third of the peritonæal coat of the organ. The same thing is sometimes observed in the spleen and liver. It is worthy of remark here, that, when the cyst is formed out of pre-existing mucous membrane, as in the instances above referred to, it generally, in the course of a short period, assumes all the properties of the serous textures."

These cysts may be regarded as secretory surfaces without excretory ducts, and hence the extraordinary dimensions to which many of them attain. By the stimulus of distension their growth is kept *pari passu* with the increase of the secreted fluid. We have seen an ovarian cyst come at last, to equal in capacity the gravid uterus at the full term of gestation. When the cyst has arrived at this magnitude, the patient exhibits all the characteristic symptoms of ascites. We have, at this time, two female patients, in each of whom an ovarian cyst has come to fill the cavity of the abdomen, with such uniformity and to such extent, that but for a reference to their early history their true anatomical history could not be known.

The study of serous cysts is interesting to the surgeon. When they are not natural cavities enlarged, but adventitious productions, they are, in all cases, manifestly allied to those semi-solid, atheromatous and steatomatous tumours, so often met with in the sub-cutaneous cellular tissue, and known under the name of wens. Even these, however, may be natural cellules, in a state of hypertrophy, reinforced in their parietes by new deposits of nutritive matter, or by the condensation of the surrounding cellular tissue. Thus an obstructed sebaceous follicle may, in the the opinion of Sir Astley Cooper, expand into an atheroma; and the fatty encysted tumours generally, may be nothing more than hypertrophied adipous vesicles.

With this subject, we conclude that part of the work before us, which treats of analogous and transformed tissues. The next chapter, divided into several sections, is devoted to the heterologous formations; the transition to which is easy and natural, inasmuch as the encysted tumours, are associated in our minds with those heterologous masses, which constitute some of the most interesting objects of surgery.

In our August No. we indulged in some speculations on the origin of these formations which we shall not now repeat. Professor Gross admits four species:—1. Tubercle, 2. Melanosis, 3. Scirrhus, 4. Encephaloid. We shall say enough under each of these heads to present the view which our author takes of them; but in the present number shall speak only

Of Tubercle. None of the heterologous formations are as frequent, as often seated in vital organs, and as fatal, as this; to which our author has, therefore, very properly devoted more space than to either of the others. He remarks with great propriety, that although not stigmatized with the epithet *malignant,* this is, indeed, one of those productions, to which that term is most appropriate; as no other more certainly or speedily effects the decomposition of the surrounding parts. Tubercle has been found in almost every tissue of the body, but, as all the world knows, especially affects the lungs. There is reason to believe, indeed, that it is never found in any of the other tissues of the adult, without being likewise found in that organ; though it often exists there when it is absent from other parts. Of the causes of this pre-eminent liability of the pulmonary apparatus, we are entirely ignorant, and, therefore, at liberty to indulge in conjectures, which for a moment we shall do. *First.* The external causes which favor the development of tubercles, may be of a kind which exert themselves chiefly on that organ. Among these causes is a cold, damp and confined atmosphere, with sedentary habits—the former acting directly on the organ, the latter indirectly, by reducing the activity of the respiratory function. *Second.* The commencement of the tubercular development may be in the blood, or in the constitution at large, which may make an effort to eliminate the tubercular matter through the lungs, when it may be deposited in the cellular tissue or air vesicles of the organ. If foreign substances are thrown into the bloodvessels, some take the direction of one excretory outlet, others another. Thus, a solution of tartarized antimony brings on diarrhœa, nitrate of potash diuresis, and phosphorus in oil, an exhalation of phosphorous acid from the bronchial tubes. But we shall not insist on these speculations.

Tubercle is deposited at every period of life, from the fœtal state to old age; but the greatest liability is from the 20th to the 40th year. In children the deposit is not so constantly in the lungs, as in adults; but has a greater disposition to affect the absorbent system and the spleen.

A great number of mammals, birds, reptiles and even insects, are subject to turbercles; impressively showing, that their causes are exceedingly pervading. Observation has rendered it probable, that in all these animals, the deposit is oftener made in the lungs than in any other part. Change to a colder climate, inactivity, confined air and innutritious diet, have appeared to be among the causes of this malady in animals. Their tubercles bear a close analogy to those of man.

The analysis of tubercle has thrown no light on its origin. Hecht of Strasburgh, found it composed of nearly equal parts of albumen, fibrin and gelatin. Thenard, on the other hand, obtained ninety-eight parts of albumen, out of a hundred. These differences may have arisen from the different stages or ages of the tubercular deposits, examined.

The most common form under which tubercular matter is deposited, is the miliary, or that which resembles millet seeds. These small bodies vary in density, from that of a semi-solid, to fibro-cartilage: and although generally of a dull yellowish colour, are sometimes gray, and at others of a milky blue.

At first perfectly distinct, the granules at length coalesce, and form large irregular masses. In general, tubercles have no other covering than the surrounding tissues: but now and then, we find them encysted. Examples of this, presented themselves in the lungs of phthisical subjects exhibited to the clinical students of the Louisville Hospital, last winter. The parietes of the cyst, none of which exceeded the size of a hazel nut, were dense, fibrous and closely adherent to the tissue in which they were embedded. Such tubercles bear a striking resemblance to those in which the peritoneal envelope of the mesenteric gland constitutes the sac.

"This variety of tubercle is very rare. Louis has seen only a solitary instance; and Professor Carswell appears altogether to doubt the possibility of its occurrence. The celebrated Laennec also met with it only on a few occasions; and, thus far, although I have made numerous examinations of persons who have died of this malady, I have never seen more than five or six well marked examples of it. The situations in which it is most commonly found are the peritonæum, the lungs, the brain, the bronchial lymphatic ganglions, and the bones. According to Meckel, the encysted variety of tubercle is more frequent in the inferior animals, as the monkey, dog, and antelope, than in the human subject."

A third variety of tubercle is the infiltrated; a fourth the stratiform. All of these varieties are represented by our author in a lithograph; and his account of each, is as full as an elementary work requires. We must bear in mind, that these distinctions are not founded on a difference in composition of the tubercular matter, or of the mode of morbid action which leads to its deposit, but on the manner of its aggregation. We cannot, therefore, attach so much practical importance to their study as many writers claim for it.

As tubercles, (not softened into a puriform fluid,) are always found more or less indurated, it has, by some pathologists been affirmed, that they are hard at the moment of their secretion. Our author ably, and as we think, successfully combats this opinion, and shows that they harden by the absorption of their thinner parts. The following extract will be read with interest:

"In certain parts of the body, as, for example, in the peritonæum, we can detect nature, as it were, in the very act of her work, and distinctly examine this substance as it is about being converted from the fluid into the solid state. In several cases of chronic inflammation of this membrane, I have discovered tubercles in every possible stage of development, some of them—evidently deposited only a day or two before the individual expired—being of a soft, viscid consistence, and perfectly transparent appearance; others semi-concrete yellowish, and consequently more or less opaque; and, lastly, another set perfectly dense and firm, like fibro-cartilage, organized, and covered by an accidental serous membrane, of the most delicate texture. Thus, the conclusion is obvious, that all tubercular matter, whatever may be its form, site, extent, is, in the first instance, of a liquid nature, and that it becomes solid only by the removal of the serosity which is always poured out simultaneously with it."

Our author has devoted a page to the nature of tubercles. We shall content ourselves with extracting its closing paragraph:

"After much reflection, however, upon the subject, and from careful and repeated examinations of tubercles in different organs of the body, in different individuals, and in different stages of their development and growth, I am constrained to believe that these heterologous formations are originally nothing but a species of coagulating lymph, thrown out as an effect of inflammatory irritation, and modified in its character according to the tissues in which it is deposited."

Having in the beginning of our review, given some reasons for dissenting from the class of pathologists, who like our author, believe in the inflammatory origin of tubercles, we shall offer no comments on his extract.

Professor Gross is an earnest advocate for the organizable constitution of tubercle. This, in fact, is but a corollary from his other proposition, that they are essentially the product of inflammatory action, and contain fibrin, the chief element of the false membranes. Not content with entrenching himself behind the authority of writers, he informs us that

"Within the last two years, I have examined not less than six specimens of organized tubercles, one occurring in the kidney, two in the spleen, one in the peritonæum, and two in the lungs. They were taken mostly from children under twelve months of age. The tubercles were of the miliary kind, and numerous vessels, loaded with florid blood, could be seen shooting into them in every possible direction, many of them penetrating a considerable distance into their substance."

These observations would undoubtedly settle the question, if it could be shown, that these vessels did not belong to or run along the processes or lamellæ of cellular tissue, enveloped in tubercular matter. We can see no reason why such enveloping should not take place. In his Organic Remains, Parkinson has figured several flocculi, or filamentous textures remaining suspended in the menstruum with which he dissolved the calcareous matter, which composed the mass of certain marine petrifactions. This delicate, lamellated structure, he regarded as the cellular membrane, of the mollusca which after death had become petrified. The stony matter had taken the place of the more perishable parts of the animal, and the cellular tissue had become enveloped and perpetuated. May not the same thing sometimes happen, in the infiltration or deposit of tuberculous matter? It may be said, however, that the cellular membrane is devoid of blood vessels. But is this the fact? Is it not *traversed* by arterial ramifications? Moreover, is it not the seat of rapid and destructive inflammation? Does not arterial exhalation go on perpetually in its areolæ? And are not these facts indicative of a degree of vascularity, sufficient to account for the appearances observed by our author?

We do not, however, regard the question of the organizable property of tubercles, as offering any great practical interest. If they become organized it is but to die and dissolve. Their organization never makes them, like the false membranes, a permanent adjunct of our organs, and we do not perceive, that in the treatment of tubercular diseases anything whatever turns upon the decision of the question. Our author admits that they do not always undergo organization; but he does not suggest any management specifically appropriate to the two conditions.

All the world knows, that tubercles soften and dissolve; that this in fact is the law of their existence, and that until this change takes place, or at least commences, they offer but little embarrassment to the organs in which they are deposited. On this process our author makes the following observations:

"But, whatever notions may be entertained concerning the origin and nature of these bodies,—whether we consider them with Wepfer, Broussais, and others, merely as diseased lymphatic ganglions, or with myself, as depositions of coagulating lymph, produced by inflammatory action, and susceptible, under certain circumstances and conditions of the system, of organization, they sooner or later become soft and fluid, the necessary consequence, it may be presumed, of their crude state. This process is supposed, by Louis and Laennec, always to begin in the centre of each mass; but Andral and Carswell maintain that it may commence at any part, indifferently, at the centre or at the circumference; and such, precisely, is the result of my own observations. As it progresses, the tubercular matter becomes daily more and more soft, moist, and unctuous, until at length it acquires the ropiness and fluidity of pus. When the degeneration involves the whole mass, it is usual to find two different kinds of matter in it; one thick, straw-colored, and inodorous, like laudable pus; the other thin, commonly tinged with blood, and mixed with small, opaque cheesy flakes. This is more particularly the case in scrofulous subjects, in whom the fluid in question often strongly resembles whey, with minute portions of curd floating in it.

"Conformably with the doctrine which it has been attempted here to enforce,—that tubercles are organized structures,—the process of softening may be supposed to be analogous to slow suppuration, by which the heterologous tissue is gradually broken up and destroyed. Let me be understood. After tubercles have existed for sometime in an organ, as, for example, the lungs, they begin to act as extraneous bodies, producing irritation in the immediately circumjacent textures. This irritation, it may be presumed, is speedily propagated to the tubercles themselves; and, as their vital endowments are comparatively feeble, and consequently incapable of much resistance, they soon yield to the invasion, the rapidity of their softening being always in direct proportion to the violence of the exciting cause and the density of the morbid growth. Those, on the other hand, who believe that tubercles are organic, maintain that their softening is brought about by the agency of the surrounding tissues, alleging that they are incapable of undergoing such a change by any powers of their own. If this explanation were correct, the process should always begin at the periphery of these bodies, which however, as was before stated, is not the case.

"The softening takes place much more rapidly in some organs than in others, in which it does either not occur at all, or only after a long period. In the lungs, where the process has hitherto been chiefly studied, it may take place as early as the end of the first month from the time of the deposition, though generally not until much later. Upon this subject, however, it is obviously impossible to lay down any definite rule, as the production of the phenomenon in question must necessarily be influenced by a great variety of causes, such as the extent of the disease, the state of the patient's health, the density of the heterologous deposit, together with numerous other circumstances which will readily suggest themselves to the mind of the reader. Occasionally the softening goes on simultaneously over a large extent of surface, so as to break down one-third, a half, or two-thirds of an organ; but this occurrence is extremely rare, and is confined exclusively to acute cases. In the lungs, the degeneration usually begins at the summit of these organs, and gradually extends towards the base, as is shown by the fact, that if these viscera be examined in this direction, we successively find, at various heights, excavations and tubercles in different stages of softening,—the more solid being almost always lowest in the scale. Before the changes, of which we have now spoken, take place, the morbid deposit appears to create little disturbance in the general economy, and may exist, sometimes to a very considerable extent, without giving rise to symptoms indicative of its presence."

With this extended extract we shall for the present number close our analytical notices. The section on tubercles running through nearly twenty pages, is replete with valuable information, and although we have questioned the soundness of some of its *speculations,* we feel it our duty in the strongest terms, to commend it to all our readers.

—

We observe that the Journals of the United States, generally, have expressed a favourable opinion of Prof. Gross's work. But the American Journal, for August, presents its readers with an extended review, the drift of which, from beginning to end, is disparagement. The means of this disparagement are the selection and array of minute imperfections, and the omission of almost every notice which might be given of rich and ample excellencies. The readers of that review, were they to rely upon its statements, could not fail to regard the work as unworthy of a perusal. The Reviewer admits that its plan is good, but insinuates that its author is behind the times, and writes like one who had made up his work in the dissecting room, in the midst of subjects, with whose history he was unacquainted. Now we affirm as a fact, that the authorities cited by Prof. Gross far outnumber those of Andral; and that they are with adequate fulness brought down to the early part of the year 1839, when the manuscript was completed. We do not, of course, intimate that *all* the facts in pathological anatomy are given, for these would fill a library; but we say, that the object of the author—the production of an *elementary* treatise, on general and special pathological anatomy—has been most ably executed. The assertion, that he has not written like a practical man, is entirely gratuitous. We do not claim for our author the opportunities of personal observation, enjoyed by

Baillie, Andral or Louis, but we refer to the work itself, for evidences of a wide and diversified experience; not a little of which has been in hospitals, but much more in private practice, in a city of 40,000 inhabitants, in which, as we believe, *post mortem* examinations are oftener permitted—indeed proposed by the friends—than in any other city of the same population in the Union. In that place Prof. G. has not only had an extensive practice; but, as we have already stated, has for several years made inspections of the kind to which we are now referring, for nearly all his brethren.

The American Journal professes to be a National Review, and often breathes a patronizing spirit. It regards itself as the official of our ancient *Alma Mater*—the organ of the American profession—*the* Messenger of the New World to the Old! We certainly have no disposition to question these high pretensions—we even feel proud of its fame, and wish it long life and wide dominion. But the sun himself has spots, and the humble inhabitants of our earth can discover and note them as blemishes. They cannot tell what occasions the dark places; nor can we exactly divine the motive which has prompted the great censor of our medical literature, to aim at the destruction, in the first year of its existence, of the first extended and systematic work on a new and difficult branch of the profession, which our country has brought forth. Nevertheless as every effect has its cause, so every act of deliberate injustice has its object, and that which, on this occasion, justice requires us to condemn, will no doubt be revealed in due time. D.

(*To be continued.*)

